# Sexual Desire, Mood, Attachment Style, Impulsivity, and Self-Esteem as Predictive Factors for Addictive Cybersex

**DOI:** 10.2196/mental.9978

**Published:** 2019-01-21

**Authors:** Nektaria Varfi, Stephane Rothen, Katarzyna Jasiowka, Thibault Lepers, Francesco Bianchi-Demicheli, Yasser Khazaal

**Affiliations:** 1 Geneva University Hospitals Geneve Switzerland

**Keywords:** sex, internet, addictive behavior, impulsivity

## Abstract

**Background:**

An increasing number of studies are concerned with various aspects of cybersex addiction, the difficulty some persons have in limiting cybersex use despite a negative impact on everyday life.

**Objective:**

The aim of this study was to assess potential links between the outcome variable cybersex addiction, assessed with the Compulsive Internet Use Scale (CIUS) adapted for cybersex use, and several psychological and psychopathological factors, including sexual desire, mood, attachment style, impulsivity, and self-esteem, by taking into account the age, sex, and sexual orientation of cybersex users.

**Methods:**

A Web-based survey was conducted in which participants were assessed for sociodemographic variables and with the following instruments: CIUS adapted for cybersex use, Sexual Desire Inventory, and Short Depression-Happiness Scale. Moreover, attachment style was assessed with the Experiences in Close Relationships-Revised questionnaire (Anxiety and Avoidance subscales). Impulsivity was measured by using the Urgency, Premeditation (lack of), Perseverance (lack of), Sensation Seeking, Positive Urgency Impulsive Behavior Scale. Global self-esteem was assessed with the 1-item Self-Esteem Scale.

**Results:**

A sample of 145 subjects completed the study. Addictive cybersex use was associated with higher levels of sexual desire, depressive mood, avoidant attachment style, and male gender but not with impulsivity.

**Conclusions:**

Addictive cybersex use is a function of sexual desire, depressive mood, and avoidant attachment.

## Introduction

### Background

The internet is widely used in everyday life, including for health-related queries [1-4] and sexual health–related purposes [5]. Cybersex is a common behavior that refers to sexually oriented Web-based activities that aim to provide erotic fulfillment or sexual gratification [6]. Cybersex includes various activities such as chatting, dating, searching for offline dates, sexual role-playing, webcam interactions, virtual reality, and pornography. These activities can be categorized as solitary-arousal (ie, watching porn), partnered-arousal (ie, chatting), and nonarousal activities (ie, sex-related information seeking) [7].

Moderate use of cybersex may contribute to the expansion of sexual knowledge and enhance offline intimate interactions and sexual communications with partners [8]. Similar to those who engage in other internet-related behaviors such as gaming [9-11], however, some cybersex users may develop addictive patterns of use with possible negative consequences [12,13]. These patterns are usually described as excessive and poorly controlled use of internet-based sexual activities that lead to problems or functional impairment and persist despite such difficulties [14,15]. No consensus has been achieved about the conceptualization of this disorder [12,16], although it is often referred to as cybersex addiction [17-20]. Nevertheless, as reported for other internet-related problem behaviors [21], it is probably an umbrella term that refers to different types of cybersex activities (solitary internet porn, sex webcams, chat, etc) and to different mechanisms (ie, positive reinforcement such as sexual gratification and arousal from porn, social rewards from chat, or negative reinforcement through escape from daily stress) [12,22,23].

Several studies have reported similarities between addictive cybersex and other addictive disorders, including reduction in executive prefrontal control (the ability to select actions or thoughts in relation to internal goals) [24], association between subjective pornographic cue-related arousal and excessive cybersex [25,26], association between striatal cue reactivity (neuroimaging showing ventral striatum activity during exposure to cybersex cues) and sexual desire [27], and subjective symptoms of cybersex addiction (feeling a loss of control in using it) [23] and patterns of positive and negative reinforcement of Web-based sexual behaviors [28]. Although it seems to be of scientific significance, research on cybersex addiction is still limited [25]. In particular, factors related to the development and maintenance of addictive cybersex remain understudied [12]. This can partly be explained by the lack of consensus about such behavioral addictions.

Possible determinants of addictive cybersex have nonetheless received preliminary attention. Sexual desire reflects the powers that draw a person toward or away from sexual behavior [29] and motivate people to sexually interact. Yet, despite the importance of sexual desire as a determinant of sexual behaviors [22,30], studies on the association between sexual desire and cybersex are still lacking. In concordance with other reports on behavioral addictions and excessive internet use [9,31], several studies on the psychopathological correlates of addictive use of cybersex frequently described an association with psychiatric disorders such as depressive moods [22]. Low self-esteem was also associated with sexting (sharing sexual photos) [32], compulsive behavior [33], and sexual addiction [34]. In addition, in agreement with other studies on addictive internet gaming [35], some studies suggested that addictive cybersex is at least partly a coping behavior that aims to regulate negative emotions [20,36].

The attachment theory argues that as a result of their childhood interactions with parents and relatives, people develop beliefs about their relations to others that come to shape their future affective, intimate, and sexual relationships and behaviors according to their attachment styles [37]. In particular, they may develop insecure attachment styles. For instance, an avoidant attachment style is linked to discomfort with close relationships, avoidance of affective commitment, and a possible increase in the search for casual interactions. In contrast, anxious attachment is related to anxiety about rejection and abandonment, possibly leading people to overengage in behaviors that aim to ensure partner availability and validation and to repeatedly check for such security [38].

Such adult attachment styles seem to influence sexual experiences, intimate relationships, and sexual behaviors and satisfaction [39]. A positive correlation was previously reported between anxious and avoidant attachment and sexual addiction [40]. Furthermore, it was [41] shown that problematic pornography use is elevated in individuals with emotional insecurities such as anxious or avoidant attachment [42] and traumatic souvenirs of the past [19].

Moreover, impulsivity is a multifaceted psychological and neuropsychological construct leading to the fulfillment of behaviors without careful anticipation [43]. Impulsivity is a transdiagnostic factor involved in addictive behaviors [44], including problem gaming [45] and internet gambling [21]. Nonetheless, to date, the association between addictive cybersex and impulsivity has also received little attention [20], and in those studies that have examined this association, mixed results were found. In some studies, lack of executive prefrontal control [25,26] and impulsivity facets were associated with addictive cybersex [25,26]. In contrast, Wetterneck et al [46] did not find any differences in impulsivity measures between addictive and nonaddictive pornography use.

A recent self-report measure of impulsivity is the Urgency, Premeditation (lack of), Perseverance (lack of), Sensation Seeking, Positive Urgency (UPPS-P) Impulsive Behavior Scale, which has been translated with stable factor structure into numerous languages [47-50]. The acronym is related to the different impulsivity facets assessed by the scale: negative urgency (the tendency to act impulsively when experiencing negative emotions), premeditation (lack of), perseverance (lack of), sensation seeking, and positive urgency (the tendency to act impulsively when experiencing positive emotions). A recent study [20] showed that negative urgency and negative affect interact in predicting addictive cybersex, whereas no other associations were found with the other impulsivity dimensions assessed, such as lack of premeditation, lack of perseverance, or positive urgency (the tendency to act impulsively when experiencing positive emotions).

Despite a possible broader conception, sexual orientation can be described as homosexuality, bisexuality, or heterosexuality [51]. In previous studies, males with a homosexual and a bisexual orientation reported differences in the use of cybersex (more frequent Web-based sexual interactions than those reported by heterosexual males) [52]. Furthermore, people in sexual minority groups, partly due to stigma, are at increased risk of health inequalities, such as addictive disorders [53] and depression [54].

### Objectives

The aim of this study was to assess the links between cybersex addiction and several psychological and psychopathological factors, including sexual desire, mood, attachment style, and impulsivity, by taking into account the age, sex, and sexual orientation (heterosexual, homosexual, or bisexual) of cybersex users. We expected to find an influence of the selected variables on cybersex addiction.

## Methods

### Recruitment Procedure

The participants consisted of users of cybersex sites and forums recruited via advertising on specialized forums and websites (pornographic sites, chat rooms, and dating sites). To be included, participants had to be more than 18 years old and to understand the languages of the questionnaires (French or English). There was no incentive for participation. The participants gave consent and then completed the questionnaires anonymously via SurveyMonkey links. The survey responses were sent over a secure—Secure Sockets Layer—encrypted connection. Internet protocol addresses were used only to check for double participation. The study did not use the participants’ names, nicknames, or email addresses, and the data were analyzed anonymously. The study protocol was approved by the Ethical Committee of the Geneva University Hospitals.

### Sample

The recruitment procedure resulted in 761 people clicking on the link to participate in the study, of whom 605 gave their consent. The participant completion rate decreased along the length of the questionnaire. Among the 605 subjects who gave their consent, 358 continued past the demographics section. Only 226 subjects continued to the last part, the questionnaire section. After missing values were removed, the final sample included 145 participants.

### Instruments

#### Compulsive Internet Use Scale

The Compulsive Internet Use Scale (CIUS) [55] consists of 14 items rated on a 5-point Likert scale ranging from 0 (never) to 4 (very often). Higher scores indicate more severe addictive use. Previous studies reported good factorial stability across time and across different samples [55]. The scale involves items related to different aspects of addictive behaviors such as loss of control, preoccupation, withdrawal, coping, and conflict. In different samples and linguistic validations of the CIUS, a 1-factor solution was repeatedly retained as the best-fit model [55-59]. The items of the CIUS ask about general use of the internet (ie, “Do you find it difficult to stop using the internet when you are online?”). To specifically assess cybersex activities, we asked participants to answer the questions while keeping in mind that the word *internet* specifically refers to cybersex use. The CIUS and other internet addiction scales have previously been successfully adapted to focus on a specific internet use to assess internet gaming, internet gambling [60], and cybersex [20,61] without alterations of their psychometric properties.

#### Sexual Desire Inventory

Consisting of 14 items on a Likert scale, the Sexual Desire Inventory (SDI) was used to evaluate sexual desire (eg, “When you first see an attractive person, how strong is your desire?”) [62].

Four items are scored from 0 (not at all) to 7 (more than once a day). The other items are answered on a 9-point Likert scale ranging from 0 (no desire) to 8 (strong desire). Higher SDI scores reveal higher sexual desire.

#### Short Depression-Happiness Scale

The Short Depression-Happiness Scale (SDHS) was used to evaluate mood variation from depressive mood (eg, “I felt dissatisfied with my life”) to happiness (eg, “I felt happy”) during the last 7-day period. It consists of 6 items, 3 positive and 3 negative, rated on a 4-point Likert scale ranging from 0 (never) to 3 (often). The lower the score, the higher the depressive symptoms [63].

#### Experiences in Close Relationships-Revised Questionnaire

This Experiences in Close Relationships-Revised (ECR-R) questionnaire was used to evaluate attachment style [64,65]. The inventory includes 18 items for anxious attachment characterized by possessive love and fear of loss (eg, “I often worry that my partner will not want to stay with me”) and 18 items for avoidant attachment characterized by fear of romantic love and low relationship success (eg, “I prefer not to show a partner how I feel deep down”). The items are rated on a 7-point Likert scale ranging from 1 (completely disagree) to 7 (completely agree). Several studies showed good test-retest reliability and a good association of the subscale scores with other ratings of daily anxiety and avoidance faced with a close companion [66].

#### Urgency, Premeditation (Lack of), Perseverance (Lack of), Sensation Seeking, Positive Urgency) Impulsive Behavior Scale Impulsive Behavior Scale

The UPPS-P Impulsive Behavior Scale [67], in its short 20-item version [47], is used to measure impulsivity according to 5 dimensions: positive urgency (strong reactions while experiencing intense positive emotions), negative urgency (strong reactions while experiencing intense negative emotions, eg, “When I am upset I often act without thinking”), lack of premeditation (tendency to disregard the consequences before acting), lack of perseverance (difficulty staying focused on a difficult or boring task), and sensation seeking. Responses are rated on a 4-point Likert scale ranging from 1 (strongly agree) to 4 (totally disagree). Good test-retest stability was previously reported [47]. In consideration of its multicomponents, the scale was of particular interest for the assessment of addictions [68]. In some studies, some of the impulsivity facets assessed with the UPPS-P, in particular negative urgency [69-72] and, depending on the assessed behaviors and sample, positive urgency [71], lack of premeditation [69], lack of perseverance [73], and sensation seeking [68], were previously associated with addictive behaviors.

#### Single-Item Self-Esteem Scale

This 1-item scale (“I have high self-esteem”) was used to measure global self-esteem [74]. Participants complete the single item on a 5-point Likert scale ranging from 1 (not very true of me) to 5 (very true of me). The Single-Item Self-Esteem Scale (SISE) showed good convergent validity with other assessments of self-esteem such as the Rosenberg Self-Esteem Scale [74]. Due to the single-item composition of the SISE, internal consistency is supposed to be perfect by definition and cannot be estimated. In this sample, this scale was normally distributed.

Age, gender (male or female), marital status (single, in a relationship—married, in a relationship—not married, widow, or widower), and sexual orientation (measured with a question asking whether the subject described himself or herself as heterosexual, homosexual, or bisexual) were also assessed.

### Analyses

Due to the small sample size for sexual orientation and marital status, demographics were compared between men and women by using the Fisher exact test, whereas the Wilcoxon rank sum test was performed for age. Regarding the different scales, when missing items represented less than or equal to 10% of all items on a specific scale (16.6% for the SDHS because it has only 6 items), the missing answer was replaced with the mean of the subject’s responses to the items on that scale (person-mean imputation). Internal consistency was assessed with Cronbach alpha [75]. To assess the variables associated with a high score on the CIUS, we performed a linear mixed model. The dependent variable was the CIUS score, and the independent variables were the SDI score, the SDHS score, the ECR-R subscales, the UPPS-P subscales, the SISE, sex, and sexual orientation. An interaction term between sex and sexual orientation was also included in the model. As there were 19 subjects who did not report their year of birth, age was not included in the model. This should not introduce bias into the analysis because the correlation between age and the CIUS score was close to 0 and did not reach statistical significance.

A linear mixed model is a statistical model containing both fixed effects, as in a classical linear regression, and random effects [76]. Random effects are useful for modeling cluster data; therefore, this type of model is suitable for correlated measurements, as it accounts for the lack of independence of the observations. In this sample, it could be assumed that subjects who filled in the French version of the questionnaire were more similar to one another than subjects who filled in the English version of the questionnaire; therefore, language was modeled as a random effect.

To determine whether the tested model was valid, we performed residual analyses and collinearity diagnostics. Residual analysis showed graphically that residuals were normally distributed, that there were no extreme values, and that they were homoscedastic. Regarding collinearity diagnostics, no variance inflation factor was higher than 4, which suggests that no collinearity problems were present [77]. Analyses were done with R 3.1.0 (R Core Team, 2014) [78]. The package nlme (R Core Team, 2017) was used to run the linear mixed model.

## Results

### Demographics of the Participants

The study involved 145 participants. When we compared the 145 included subjects with those who at least provided their age, sex, and sexual orientation, no statistical differences were found.

Table 1 shows the demographics of the participants. The sample was composed of 60.0% (87/145) men and 40.0% (58/145) women. The median age of the sample was 31 years (range: 18-70 years). Women were younger than men (28 years vs 36.5 years, respectively, *P*=.014). Regarding marital status, 37.9% (55/145) of the participants were single, 39.3% (57/145) in a relationship—not married, 20.7% (30/145) in a relationship—married, and 2.1% (3/145) widows or widowers. Sexual orientation and sexual orientation within sex were also measured: 77.9% (113/145) of the participants reported being heterosexual, 7.6% (11/145) being homosexual, and 14.5% (21/145) being bisexual. Among men, 79% (69/87) reported being heterosexual, 6% (6/87) being homosexual, and 13% (12/87) being bisexual; among women, 75% (44/58) reported being heterosexual, 8% (5/58) being homosexual, and 15% (9/58) being bisexual.

**Table 1:** Demographics of the participants.

Characteristic		Whole sample	Women (n=58)	Men (n=87)	*P* value
Age, median (range)		31 (18-70)	28 (18-70)	36.5 (18-70)	.014^a^
**Sexual orientation^b^, n (%)**					0.87
	Heterosexual	113 (77.9)	44 (38.9)	69 (61.1)	
	Homosexual	11 (7.6)	5 (45.5)	6 (54.5)	
	Bisexual	21 (14.5)	9 (42.9)	12 (57.1)	
**Marital status^c^, n (%)**					0.49
	Single	58 (40.0)	21 (36.2)	37 (63.8)	
	In a relationship	87 (60.0)	37 (42.5)	50 (57.5)	

^a^W statistic for the Wilcoxon rank sum test is 2500.5.

^b^Women/men proportions are within sexual orientation categories.

^c^Women/men percentages are within marital status categories.

### Instruments

Table 2 shows the means and SDs of the instruments used as well as Cronbach alpha [75] as a measure of internal consistency and its 95% confidence interval. Every instrument had good (>0.80) to excellent (>0.90) internal consistency, but the UPPS-P positive urgency scale fell into the acceptable range (>0.70).

### Results of the Linear Mixed Model

The results of the linear mixed model are reported in Table 3. The most important influences on the CIUS scores (see standardized coefficients) were lower SDHS scores (meaning more depressive scores), followed by higher avoidant attachment style scores, male gender, and higher sexual desire. The other variables (anxious attachment, UPPS-P subscales, SIUS, sexual orientation, and interaction between gender and sexual orientation) did not reach statistical significance on the CIUS scores.

**Table 2:** Description of the instruments.

Instrument		Mean (SD)	Cronbach alpha	95% CI
Compulsive Internet Use Scale		14.64 (9.84)	.89	0.89-0.91
Sexual Desire Inventory		70.83 (17.66)	.87	0.84-0.90
Short Depression-Happiness Scale		11.29 (4.38)	.86	0.83-0.90
**Experiences in Close Relationships-Revised questionnaire**				
	Anxious attachment	3.39 (1.33)	.92	0.91-0.94
	Avoidant attachment	3.07 (1.04)	.89	0.86-0.91
**UPPS-P^a^ Impulsive Behavior Scale**				
	Positive urgency	10.44 (2.57)	.74	0.67-0.81
	Negative urgency	8.64 (3.04)	.86	0.82-0.89
	Lack of premeditation	7.45 (2.64)	.80	0.75-0.85
	Lack of perseverance	7.34 (2.66)	.84	0.80-0.88
	Sensation seeking	11.31 (2.70)	.80	0.74-0.85
Single-Item Self-Esteem Scale		2.61 (0.83)	—^b^	—

^a^Urgency, Premeditation (lack of), Perseverance (lack of), Sensation Seeking, Positive Urgency.

^b^Not applicable.

**Table 3:** Results of the linear mixed model.

Characteristics and measures		Regression coefficient	Standard error	*t* value (degrees of freedom)	*P* value	Standardized coefficients
Female versus male		−3.82	1.75	−2.18 (128)	.03	−0.19
**Sexual orientation (reference group: heterosexual)**						
	Homosexual	0.08	3.67	0.02 (128)	.98	0.07
	Bisexual	−1.37	2.61	−0.52 (128)	.60	0.10
**Interaction (female)**						
	Homosexual	1.62	5.58	0.29 (128)	.77	0.08
	Bisexual	5.81	4.13	1.41 (128)	.16	0.29
Sexual Desire Inventory		*0.11* ^a^	*0.04*	*2.48* (128)	*.01*	*0.19*
Self-esteem		−0.68	1.00	−0.67 (128)	.50	−0.06
**UPPS-P^b^ Impulsive Behavior Scale**						
	Positive urgency	0.19	0.33	0.57 (128)	.57	0.06
	Negative urgency	−0.15	0.37	−0.39 (128)	.69	−0.04
	Lack of premeditation	0.31	0.34	0.92 (128)	.35	0.08
	Lack of perseverance	−0.07	0.36	−0.20 (128)	.84	−0.02
	Sensation seeking	0.07	0.30	0.25 (128)	.80	0.02
Short Depression-Happiness Scale		− *0.85*	*0.22*	− *3.95* (128)	*>.001*	− *0.38*
**Experiences in Close Relationships-Revised**						
	Anxiety	−0.56	0.70	−0.81 (128)	.42	−0.08
	Avoidance	*2.20*	*0.79*	*2.79* (128)	*.006*	*0.23*

^a^Italics represents significant regression parameters.

^b^Urgency, Premeditation (lack of), Perseverance (lack of), Sensation Seeking, Positive Urgency.

## Discussion

### Principal Findings

The aim of this study was to study cybersex addiction and to assess the links between cybersex addiction and possible determinants of such behavior, namely, sexual desire, mood, attachment style, and impulsivity, by taking into account the age, sex, and sexual orientation of cybersex users. We concluded that addictive cybersex use, as assessed by the CIUS adapted for sexual activities, is associated with sexual desire, depressive mood, an avoidant attachment style, and male gender. As shown in Table 3 (standardized coefficients), the results suggest that the most important influence on the CIUS scores is depressive mood, followed by avoidant attachment style, male gender, and sexual desire. UPPS-P impulsivity subscores, self-esteem, and sexual orientation do not have a significant influence on addictive cybersex.

Sexual desire is an important drive for sexual behavior and is positively associated with emotional intimacy [79]. In this study, elevated sexual desire was significantly associated with addictive cybersex use. This finding is consistent with the gratification hypothesis [26] and with previous findings showing an association between cybersex use and arousal and craving for specific porn cues [80]. The results suggest that at least part of addictive cybersex use is linked to such positive reinforcement. Sexual desire is also known for its modification related to depressive mood [81]. Possible fluctuations between sexual desire, mood modification, and cybersex use could be assessed in future studies by using methods that are based on ecological momentary assessment [82].

Our finding of an association between addictive cybersex use and depressive mood is congruent with other studies that showed the importance of links between addictive cybersex and diverse assessments of psychological distress and mood [22,26]. This finding is also in line with other reports of the association between excessive internet gaming [83] or internet gambling [21] and depressive mood. Such associations suggest that addictive cybersex is at least partly a coping behavior that aims to regulate negative emotions [20,35,36,84]. This finding opens the debate, as has occurred for other internet addictive-like behaviors, about an appropriate diagnostic framework [16] and adequate understanding of such an association [85]. The possible development of psychopathological distress, which could lead to a more pronounced depressive mood secondary to the negative impact of addictive cybersex (interpersonal isolation and reduction of offline sexual activities), cannot be ruled out [86], and thus, further prospective studies are warranted.

We also found an association between addictive cybersex use and avoidant attachment but not anxious attachment. These results are congruent with those of other studies showing the implications of insecure attachment in excessive internet use [19] and cybersex [41]. Beutel et al [42] found an increase in the intensity of internet sex use with the importance of anxious attachment. Their results failed, however, to reach statistical significance for the link between the importance of internet sex use and avoidant attachment. Such differences could possibly be explained by differences in cybersex use assessment methods. In fact, Beutel et al’s study used more items related to cybersex use (eg,“ I have searched for sexual materials online...”) and only 2 items related to addictive cybersex (ie, “I believe that I am an internet sex addict” and “I have promised myself to stop using the internet for sexual purposes”). Furthermore, items were on a dichotomous scale (true or false), which may limit the ability to detect variability. The association found with avoidant attachment could be explained by displeasure and fear of close relationships, which lead to an increase in cybersex activities that less often involve closeness in relationships. In this study, the lack of association between addictive cybersex and anxious attachment style was possibly because of the limitations in sample size. One could hypothesize differences in attachment style across specific cybersex activities (ie, anxious attachment may have more Web-based interactions with potential partners because of anticipated fear of rejections). Further studies should assess specific cybersex activities in more detail. Despite such differences across studies, insecure attachment styles play an important role in cybersex addiction. As suggested elsewhere [19], such findings deserve clinical investigation and treatment of attachment style for patients who are involved in addictive cybersex.

Impulsivity and cybersex addiction were not significantly associated in our study. The results of the study at hand contrast with those of other studies regarding the links between the UPPS-P and internet-related addictive behaviors [21,45]. The results of this study are contrary to those of previous studies showing some associations between addictive cybersex and impulsivity [20,46]. Furthermore, using the same UPPS-P scale, Wery et al [20] showed that in a group of male participants, negative urgency interacted with negative affects in predicting addictive cybersex. However, the strength of the association was not strong, as shown by the authors’ reported odds ratio of 1.03 (95% CI=1.01-1.06). In another study, Wetterneck et al [46] showed a small correlation between a measure of impulsivity and the number of hours of porn use by week. However, they did not report significant differences in impulsivity between a group of addictive porn users and controls.

In light of such observations across studies, one may hypothesize that some impulsivity facets may contribute to addictive cybersex without having a main determinant effect on such behavior. This may contribute to disparities between studies. Furthermore, such differences are possibly influenced by sample size, the specific type of cybersex activities (ie, possible differences between porn use and sex dating), and other assessments involved in the analyses. For instance, our study included measures of attachment, a construct not included in the previously mentioned studies. However, we cannot exclude the possibility of modifications in executive functions when an individual faces specific cybersex cues [24] or during interactions with negative states and cybersex use [20]. Further studies on the possible role of impulsivity constructs in addictive cybersex are needed.

Self-esteem had no impact on CIUS scores. This result contradicts those of other studies that show, for instance, an association between low self-esteem and adolescent sexting (sharing sexual photos) [32]. These differences between studies may be because of sample characteristics, participants’ specific cybersex activities, or the assessment methods. This study, for example, assessed general self-esteem with only 1 question. Furthermore, the impact of specific cybersex activities on self-esteem cannot be ruled out. Prospective studies on the links between such activities and self-esteem, including possible mediators of effects such as fear of negative evaluation [33], are needed.

This study also showed an association between addictive cybersex and male gender, as has repeatedly been found [17,42,46,87,88]. Sociocultural differences may contribute to this phenomenon. Moreover, possible differences between men and women in sexual desire, sexual arousal, and their interplay may contribute to the observed difference [89]. The design of sex-related websites and mobile phones apps may also influence gender differences in cybersex use. Gender differences were commonly reported in addictive disorders; additional studies are required to understand the underlying mechanisms [90].

Among a population of cybersex users, our study showed no association between age and cybersex addiction. Most studies on cybersex have involved adolescents and young adults [17]. Some earlier studies (in the early 2000s), however, showed that adults older than 50 years were less prone to cybersex use than younger adults [91]. The findings of this study are possibly explained by a focus on cybersex addiction (and not on cybersex use) and by societal evolution and wider access to the internet in all age ranges.

In this study, sexual orientation had no effect on the assessed behavior. Similarly, no effect was found in the interactions between gender and sexual orientation. However, sexual orientation was assessed in only 3 main categories (heterosexual, bisexual, and homosexual). Future studies would benefit from more refined evaluations of sexual orientation [51] and its possible components (eg, erotic fantasy and social interactions) [92] as well as from evaluations of gender identity and its related distress [93].

Cybersex is associated with addictive use for only a small number of users [20]. This observation is also illustrated by the mean (Table 2) and median (13 of 56) of the CIUS scores in this study. Nonetheless, for those with addictive patterns of use, treatment options are still sparse and understudied; most of the few preliminary studies in the field have tried to reproduce what is already known from the psychotherapy of addictive disorders [12].

The findings of this study have clinical implications. It seems important to consider cybersex addiction in terms of its principal connections with several psychological dimensions. Particular attention should be given to the patient’s patterns of attachment. Psychotherapeutic treatment has to be tailored to the specific needs of each patient. People with avoidant attachment, for example, may benefit from a psychotherapeutic approach designed to integrate treatment of addiction and attachment disturbances. Future studies for the assessment and treatment of cybersex addiction are needed in clinical settings.

### Limitations

Several limitations of the study must be considered. The sample was relatively small but adequate for the study statistics. Furthermore, the sample was exposed to self-selection biases [94]. The cross-sectional design did not allow assessment of longitudinal interplay between the assessed variables. Furthermore, the study did not take into consideration the different cybersex activities that could influence cybersex use across different behaviors and cybersex communities. Finally, there is no consensus related to cybersex addiction, and thus, the study used the CIUS adapted to cybersex as a proxy. Using a continuous approach rather than a categorical one, however, allows assessment of some determinants of the severity of addictive cybersex use with an adequate research instrument related to addictive use of internet-delivered services.

### Conclusions

Despite these limitations, this study indicates that addictive cybersex is influenced by an avoidant attachment style, depressive mood, and sexual desire. Males are at increased risk. Self-esteem and impulsivity do not seem to have a significant influence on addictive cybersex. Further research, including prospective studies, is needed in the field.

## Abbreviations

CIUS: Compulsive Internet Use Scale
ECR-R: Experiences in Close Relationships-Revised
SDHS: Short Depression-Happiness Scale
SDI: Sexual Desire Inventory
SISE: Single-Item Self-Esteem Scale
UPPS-P: Urgency, Premeditation (lack of), Perseverance (lack of), Sensation Seeking, Positive Urgency) Impulsive Behavior Scale
